# Synthetic plant disease image generation to improve segmentation tasks in low-resource settings

**DOI:** 10.3389/fpls.2026.1812323

**Published:** 2026-05-08

**Authors:** Elliott Cooper, Zane Hartley, Andrew French

**Affiliations:** School of Computer Science, University of Nottingham, Nottingham, United Kingdom

**Keywords:** ControlNet, leaf segmentation, low-rank adaptation, low-resource learning, plant disease detection, semantic segmentation, stable diffusion, synthetic data

## Abstract

Accurate plant disease segmentation is often constrained by the availability of large, finely annotated datasets, particularly for rare diseases. This work presents a synthetic data generation pipeline that combines 3D leaf modelling with diffusion-based disease synthesis to address this limitation. Procedurally-generated leaf geometries are built in the 3D modelling package Blender to provide exact ground-truth masks, after which style-transfer is applied using Stable Diffusion, fine-tuned with Low-Rank Adaptation (LoRA) and guided by ControlNet conditioning to both preserve leaf structure and enforce correct lesion placement. The approach is evaluated on apple leaf diseases using a deliberately restricted subset of the PlantVillage dataset, simulating a controlled low-data-resource environment. Downstream task effectiveness is measured through leaf disease segmentation. The results show that combining data from the pipeline with limited real data leads to consistent improvements in segmentation performance.

## 1 Introduction

Plant disease detection is a critical application of computer vision in agriculture, enabling early diagnosis and prevention of crop loss. Deep learning-based image segmentation approaches have shown promise in identifying disease regions in plant leaves; however, these methods typically require large annotated datasets that are costly and time consuming to create, particularly for rare diseases. This work addresses the limitations of low-resource datasets for segmentation of apple tree leaves, affected by three types of disease: *Botryosphaeria obtusa* (black rot), *Gymnosporangium juniperi-virginianae* (cedar rust) and *Venturia inaequalis* (scab).

Agricultural productivity is under increasing strain, with up to 40% of global crop production lost annually due to plant diseases, posing a major threat to global food security (Food and Agriculture Organization of the United Nations, 2025a). Climate change further exacerbates this pressure: rising temperatures and altered precipitation patterns disrupt plant defence mechanisms and facilitate pathogen spread (Kumar et al., 2024). The socio-economic consequences are particularly severe in low-resource agricultural systems, where limited access to diagnostic technologies restricts early intervention (Food and Agriculture Organization of the United Nations, 2025b). Despite the urgent need for scalable disease detection systems, there remains a shortage of high-quality annotated datasets, especially for rare diseases and low-resource environments.

Automated phenotyping leverages computer vision to enable rapid disease identification at scale. High-throughput imaging modalities, including RGB (Fahlgren et al., 2015), multispectral (Mahlein, 2016) and hyperspectural systems (Mahlein et al., 2018), allow detailed capture of plant health. However, reliable segmentation models require large, diverse and densely annotated datasets. In practice, annotation is labour intensive, slow and often inconsistent, particularly for leaf diseases where symptoms vary in appearance and severity. This disparity between rapid image acquisition and slow manual annotation forms the phenotyping bottleneck (Furbank and Tester, 2011), limiting progress in agricultural computer vision.

Field data collection presents further challenges. Illumination variability, occlusion from overlapping foliage, seasonal constraints and geographic specificity introduce inconsistencies in real-world datasets. Benchmark datasets such as PlantVillage demonstrate strong in-domain performance, yet capture a narrow subset of environmental variability (Barbedo, 2019). Models achieving 85–99% accuracy on PlantVillage images have been shown to degrade to 31% when evaluated on field data (Mohanty et al., 2016), highlighting the difficulty of generalising from controlled laboratory conditions to in-field environments. These limitations create a persistent performance gap between controlled and real-world settings.

Biological variability compounds this challenge. Leaf morphology varies substantially across species and genotypes, influenced by both genetic and environmental factors. A large-scale analysis of over 9,000 apple leaves from 869 variants demonstrated significant variation in aspect ratio and shape complexity (Migicovsky et al., 2018). Leaf surface properties, including wax density and wettability, introduce further textural diversity (Huber and Gillespie, 2003). Such biological heterogeneity is rarely captured comprehensively in limited datasets, reducing model robustness and generalisability.

Since the emergence of deep learning methods, architectural advances have improved segmentation performance. Encoder–decoder networks such as U-Net (Ronneberger et al., 2015) combine contextual and fine-grained features through skip connections and have become standard in plant segmentation tasks. Variants such as U-Net++ (Zhou et al., 2018) reduce semantic gaps between encoder and decoder representations at the cost of increased complexity. Vision Transformers (Dosovitskiy et al., 2021) capture long-range spatial dependencies but depend heavily on large-scale pretraining (Zou et al., 2025), limiting applicability in low-resource settings. Multi-stage pipelines integrating localisation, classification and segmentation can achieve high reported accuracy under controlled conditions (Polly and devi, 2024), yet often suffer from increased computational complexity and limited scalability.

Synthetic data generation has emerged as a promising strategy to address dataset scarcity. Conventional augmentations improve robustness but cannot introduce new pathological structures. Generative Adversarial Networks (GANs) (Goodfellow et al., 2014) were amongst the first approaches to synthesise plant disease imagery, with dual-discriminator architectures improving classification accuracy by approximately 7.5% on PlantVillage (Wang et al., 2025). However, GAN-based methods frequently exhibit training instability and visible artefacts in fine lesion regions.

Procedural 3D pipelines offer precise control over geometry, lighting and ground-truth masks. Blender (Blender Foundation, 2024) based synthetic datasets have achieved Dice scores exceeding 94% in downstream segmentation tasks (Aghamohammadesmaeilketabforoosh et al., 2025). Nevertheless, *purely* 3D renderings often exhibit domain shift due to mismatches in texture realism and biological variability (Ben Salem, 2024). Hybrid approaches combining 3D geometry with diffusion-based style-transfer have improved realism and downstream performance relative to CycleGAN-based methods (Hartley et al., 2024), though prior work remains constrained to simplified plant morphologies.

Diffusion models offer a compelling way to improve the realism of model-based images further still. (Rombach et al., 2022) generate high-fidelity imagery via iterative denoising in latent space and have surpassed GANs in fine detail reproduction. Conditioning mechanisms such as ControlNet (Zhang et al., 2023) enable spatial guidance during generation (i.e. we can keep the boundary of the leaf or disease lesion intact, allowing accurate annotations), whilst Low-Rank Adaptation (LoRA) (Hu et al., 2021) allows efficient domain-specific fine-tuning of large diffusion models (i.e. we can produce realistic styletransfer without many example images). These advances enable controllable, high-resolution synthetic data generation with reduced computational cost. However, existing inpainting-based or loosely-conditioned pipelines are not well suited to plant disease synthesis, where lesions are sparse and require precise pixel alignment.

Here, we develop and validate a synthetic data generation pipeline integrating procedural 3D leaf modelling with conditioned diffusion and disease-specific LoRA fine-tuning to improve segmentation performance under limited real-data conditions. Specifically, this work: (i) develops a hybrid synthetic pipeline combining Blender modelling, ControlNet conditioning and LoRA adaptation for realistic diseased leaf generation; (ii) evaluates segmentation performance across real-only, synthetic-only and combined training regimes; and (iii) analyses distributional similarity and semantic alignment to quantify realism. We hypothesise that spatially aligned, visually plausible synthetic images can mitigate data scarcity effects and enhance downstream segmentation accuracy in low-resource settings when combined with limited real data.

## 2 Materials and methods

**Figure 1:**
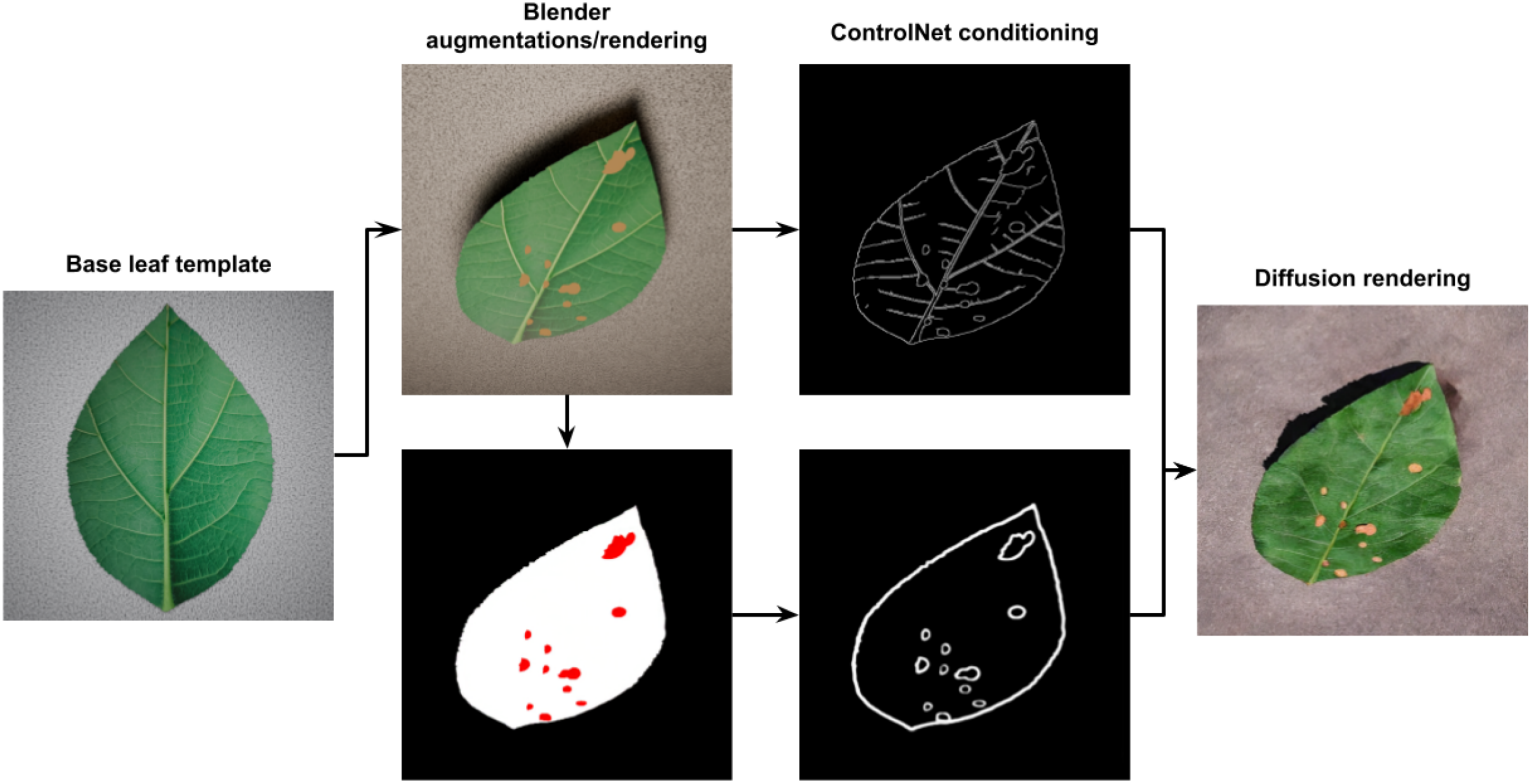
End-to-end overview of the proposed pipeline. A base leaf template is augmented in Blender to produce structural variations and lesion masks, which are converted into ControlNet conditioning signals. These guide the diffusion model to generate realistic diseased leaf images with aligned lesion structure.

The synthetic data generation pipeline is described here. 3D leaf models are first generated to provide structural guidance and ground-truth masks. A diffusion model is then applied to synthesise realistic diseased images. Multiple ControlNets enforce pixel-level alignment during generation, whilst LoRA enables accurate style-transfer. An overview of the full pipeline is shown in **Figure 1**. We describe each step of the pipeline in detail below.

### 2.1 Dataset

**Figure 2:**
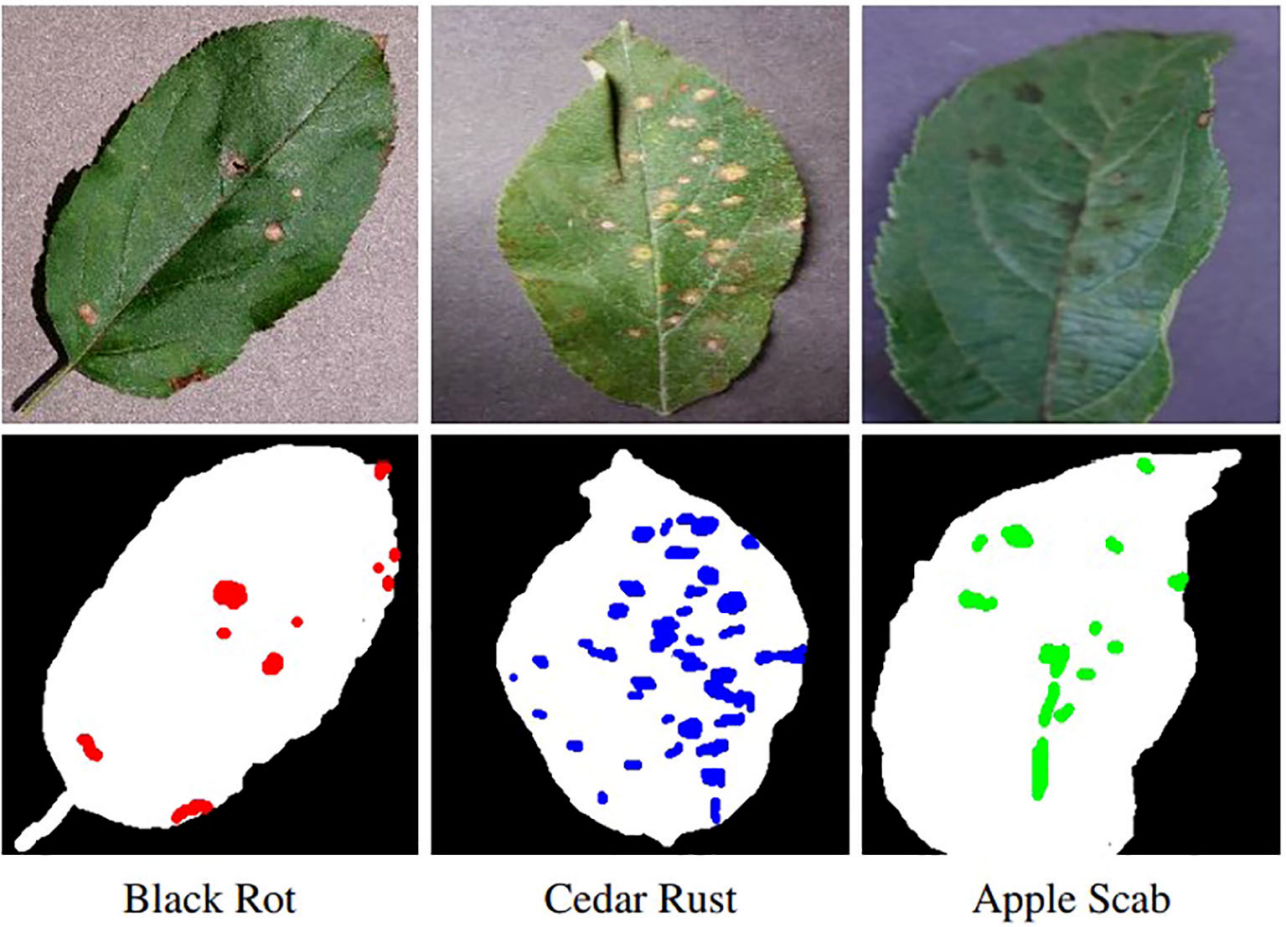
Diseased leaf sample for each disease from the PlantVillage dataset (top row) with the corresponding manually annotated segmentation masks (bottom row).

PlantVillage (Hughes and Salathe, 2016), a high-quality benchmark dataset was chosen and manually restricted to remove structural bias and poor-quality images. Three disease classes were chosen to ensure a sufficiently challenging downstream task and to evaluate the pipeline across distinct disease characteristics. Apple leaves from the PlantVillage dataset with black rot, cedar rust and scab were selected due to strong class representation, diverse leaf appearance and varying disease severity. These diseases were primarily localised lesions, making them well suited to controlled synthetic modelling, whilst laboratory capture conditions allow the pipeline to focus on disease synthesis and spatial alignment without background complexity. A sample from each disease is illustrated in **Figure 2**.

Twenty five images were selected from each disease class to form the restricted dataset, providing sufficient visual variability whilst reflecting low-resource conditions. This simulates a real-world experimental setting, where we want to phenotype a small number of diseased leaves but we don’t have access to a larger dataset for pre-training, or access to a pre-trained model due to the specific leaf/disease combination present. To note, the PlantVillage dataset only contains classification labels for each image, therefore masks were manually annotated. Computer Vision Annotation Tool (CVAT) was used due to its strong support for fine pixel-level annotation making it ideal for plant diseases, particularly where fine boundaries and overlapping classes need to be represented accurately. This annotated subset of PlantVillage is available at https://doi.org/10.5281/zenodo.18659728. The repository contains the 75 images from the restricted dataset with the corresponding segmentation masks along with 100 synthetic images per disease generated using Blender and Stable Diffusion, each with corresponding ground truth masks.

### 2.2 Synthetic leaf modelling

Synthetic leaf models are generated using Blender 4.5 (Blender Foundation, 2024), selected for its precise control over geometry, materials and lighting, enabling realistic plant structure replication with exact ground-truth masks. Three representative leaf images were selected from the PlantVillage dataset as base geometric templates. The reference leaves are morphologically representative yet geometrically simple (minimal damage or deformation) to support controlled augmentations. The images were used solely as visual references during manual mesh construction in Blender. Each reference leaf was modelled using three surface components. A shared base texture provided the RGB colour map defining overall pigmentation and venation appearance. A bump map was applied alongside the texture to simulate irregularities and improve visual depth without increasing mesh complexity. A displacement modifier was added to the perimeter of the leaf to introduce sharp and natural edge variations with a clouds texture to generate fine-scale irregularities. Laboratory capture conditions in the PlantVillage dataset were approximated using a Gaussian noise plane and uniform area lighting to provide a bench-like background to the leaves.

Blender’s Python API enables automated model generation, augmentation and rendering, which is essential for large scale synthesis. Augmentations, described in **Table 1**, were applied to the base model to reflect the natural variability observed in the dataset. Geometric, camera and lighting augmentations were applied within constrained parameter ranges chosen to mirror the limited viewpoint and capture variability observed in the PlantVillage dataset, whilst avoiding unrealistic transforms. Background texture parameters were also varied to reduce overfitting to a fixed render configuration.

**Table 1:** Geometric, camera and lighting augmentations applied during Blender-based synthetic leaf generation.

Random augmentation	Parameter range
Z-axis rotation	± 0.25*π* radians
Uniform scaling	0.8–1.2×
Translation (X, Y)	*x* ± 0.2, *y* ± 0.3
Camera tilt (X-axis)	± 0.25*π* radians
Light power	0.5–3.0× original intensity
Light colour temperature	2500–8500K
Light position (X, Y)	± 1.0 units
Light position (Z)	+1.0–2.5 units

**Figure 3:**
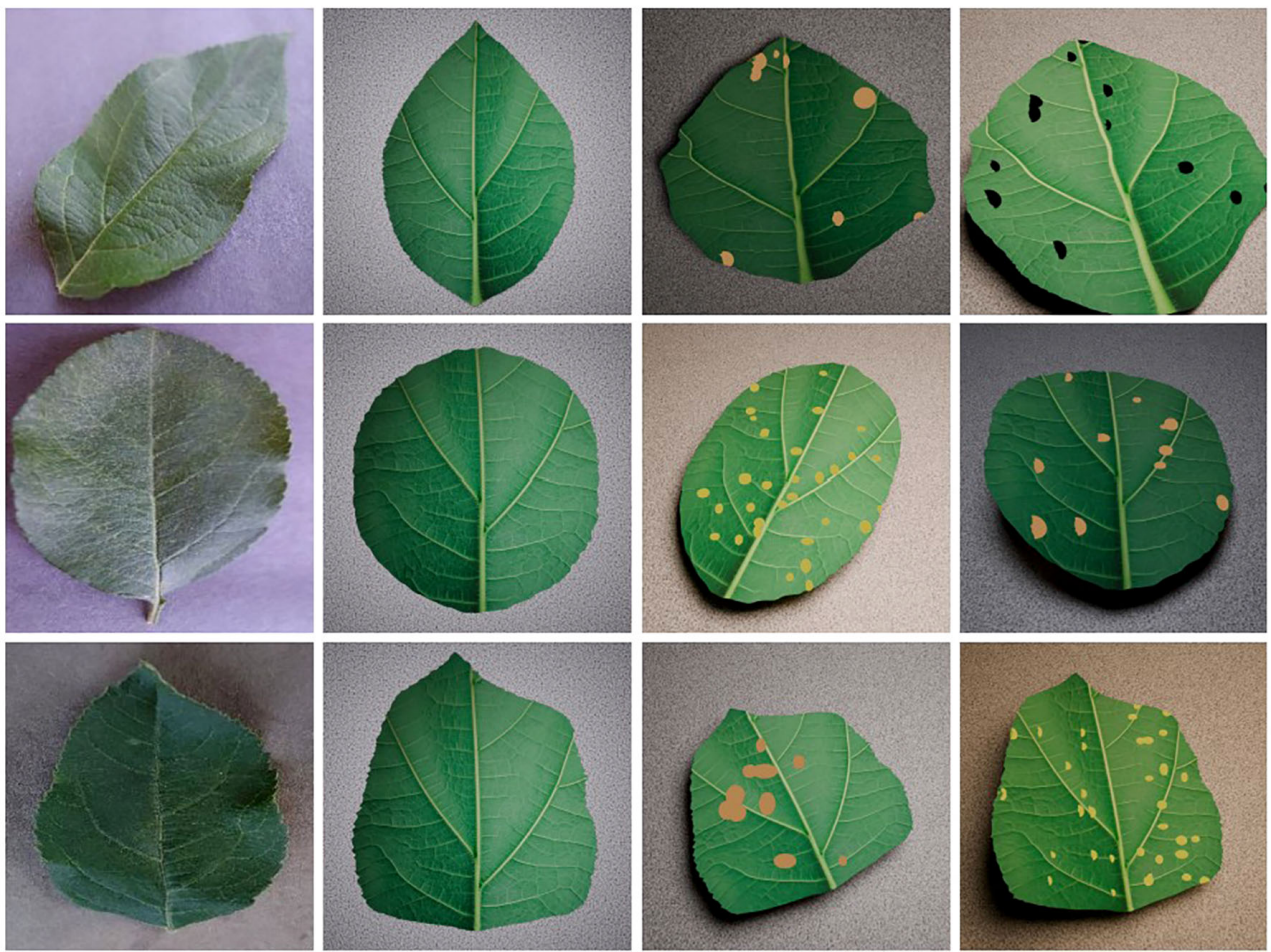
Blender synthetic leaf pipeline showing reference leaves (column 1), base 3D models (column 2) and sample augmented outputs (column 3-4).

Furthermore, procedural deformations introduce controlled variation in leaf curvature and overall proportions, increasing silhouette and boundary diversity beyond simple scaling or rotation. Disease markers were created automatically by generating spheres at randomly sampled surface locations on the leaf mesh. Marker count and size were varied by disease class: Cedar rust (16–35 spheres, radius 0.04–0.06), scab (2–15 spheres, radius 0.08–0.12) and black rot (2–15 spheres, radius 0.05–0.12), reflecting differences in typical lesion density and morphology. Each sphere was constrained to the leaf surface so that it deformed consistently with subsequent geometric augmentations. An overview of the outputs from this Blender pipeline is shown in **Figure 3**. Overall, this step allows us to generate many variations from a single base model. This process is designed to capture the visual characteristics of disease patterns rather than strict biological accuracy, enabling broader adaptability to other conditions at the cost of disease-specific realism.

Rendering was performed using the Cycles engine to achieve physically based shading and illumination. RGB images and segmentation masks were generated at 512×512 resolution, matching the native resolution of Stable Diffusion v1.5 and allowing Blender outputs to be used directly as initial images for style-transfer. Segmentation masks were produced in a separate render pass using flat emission materials with shading, shadows and colour management disabled. The leaf surface was assigned a uniform white material, whilst disease markers were rendered using disease-specific RGB colour encoding. This ensured pixel-exact class separation without lighting artefacts, enabling direct use of the masks for downstream conditioning and evaluation.

### 2.3 Low-rank adaptation

LoRA (Hu et al., 2021) is used in the pipeline to adapt Stable Diffusion to disease-specific characteristics whilst preserving its general image generation capabilities. A separate LoRA was fine-tuned for each disease class, enabling each model to learn disease-specific lesion characteristics without interference from similar disease characteristics.

For training, 15 images per disease are used (which is, to note, a very limited, low-resource dataset), with the remaining 10 images held out for downstream segmentation evaluation. Each image is annotated with captions describing lesion location and appearance, along with a disease-specific trigger token. Fine-tuning is performed at 256 × 256 to match the resolution of the PlantVillage dataset, avoiding interpolation artefacts and allowing lesion-level texture and boundary features to be correctly learned. All LoRA models are fine-tuned from Stable Diffusion v1.5 for 1000 training steps on a single NVIDIA A100 GPU partition with 16 GB VRAM.

### 2.4 ControlNet

**Figure 4:**
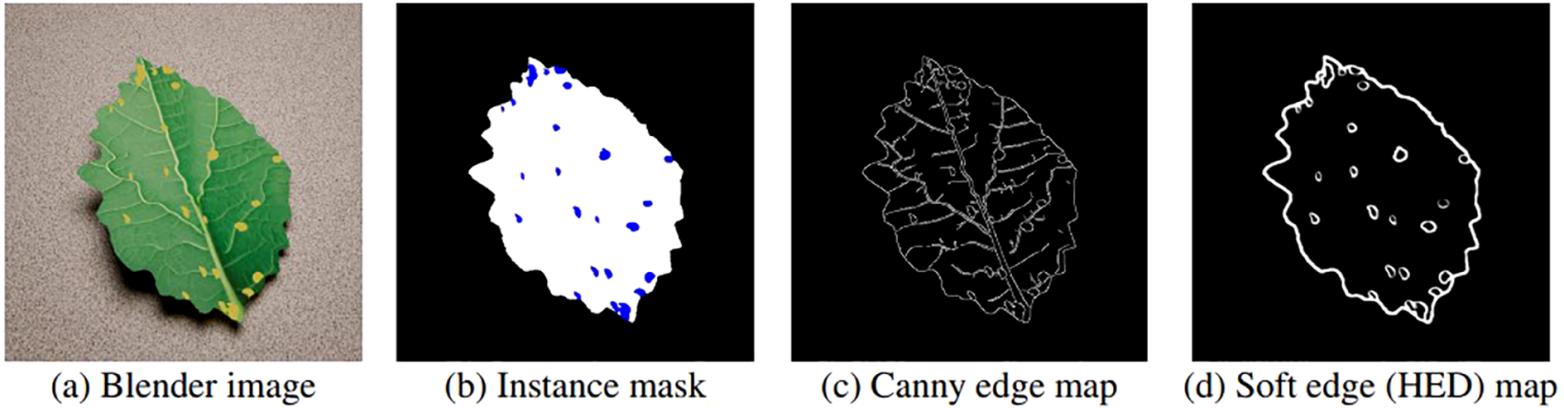
Conditioning inputs used during diffusion synthesis: **(a)** Blender render, **(b)** instance mask, **(c)** Canny edge map and **(d)** SoftEdge (HED) map.

To achieve pixel-perfect synthesis, making sure the diffusion approach does not affect the geometry of the images, two ControlNet architectures, *Canny Edge* and *Soft Edge*, are used to condition the diffusion process. using two input images: the RGB Blender render (**Figure 4a**) and its corresponding segmentation mask (**Figure 4b**). The Canny Edge ControlNet is applied to the Blender generated RGB image (**Figure 4c**) to extract high contrast gradients. This produces a binary map of the leaf’s fundamental features and geometry, such as shape outline, vein pattern and disease location, ensuring structural boundaries remain intact during the de-noising process. In addition, the Soft Edge model utilises a Holistically-Nested Edge Detection (HED) network (Xie and Tu, 2015) to identify shapes and boundaries and is applied to the instance mask (**Figure 4d**). This provides a smooth and detailed guide for specifically focusing on disease lesions, ensuring the placement on the output is correct.

### 2.5 Style-transfer

The style-transfer pipeline is implemented using the Diffusers library (von Platen et al., 2022), chosen for its well-documented Stable Diffusion and ControlNet implementations, as well as built-in support for LoRA fine-tuning via established training scripts. Stable Diffusion v1.5 is used as the base model due to its strong performance and memory-efficient inference at the target resolution of 512 × 512. The diffusion model parameters, outlined in **Table 2**, were selected through empirical tuning, guided by both quantitative segmentation performance and qualitative visual inspection. For each disease, the model is adapted using a disease-specific LoRA and prompted with the respective trigger token. A 3D leaf model containing lesion seed markings is used as the input image. For each input image, a HED edge map from the instance mask and a canny edge map from the RGB image are extracted and used to condition the LoRA. The output is a style-transferred image with the pixel-aligned mask from Blender.

**Table 2:** Stable diffusion generation parameters.

Parameter	Value
Control scale (black rot, cedar rust)	[0.7, 0.7]
Control scale (scab)	[1, 1]
Inference steps	30
Guidance scale	5
Strength	0.7
Image size	512 × 512

### 2.6 Segmentation model

We test the effectiveness of the image dataset generation by evaluating the impact on a downstream segmentation task. Deep learning architectures such as U-Net (Ronneberger et al., 2015) and DeepLab (Chen et al., 2018) have become the standard for segmentation tasks. Both models are capable of achieving strong performance, yet they differ in complexity, computational cost and suitability for different types of datasets. U-Net was chosen as it better suits the characteristics of plant disease detection. Its skip connections retain feature information, making it more effective at identifying small, sharply bounded lesions that appear in leaves. U-Net also typically has fewer parameters, requiring less computational demand and therefore a quicker training time. The ResNet-34 encoder was jointly selected as the encoder as it has approximately 21 million parameters, providing enough capacity to capture the complex disease patterns on leaves without overfitting.

Lesion regions are small, irregular and severely under-represented within an image, causing pixel-level losses to be dominated by majority classes. To help with this, a weighted Dice loss function (**Equation 1**) is used which optimises region-level overlap and is less sensitive to minor boundary misalignments. The weights are defined such that $w_{c}\propto 1/\sqrt{f_{c}}$, where $f_{c}$ denotes the pixel frequency of class c, with weights normalised across classes. The final loss is therefore

(1) $$L_{WD}=1-\frac{1}{C}\sum_{c=1}^{C}w_{c}\;{\text{Dice}}_{c}.$$

### 2.7 Runtime and training cost

For practical feasibility of the pipeline, approximate runtime and training costs are reported under the experimental configuration. Image generation using the diffusion pipeline (including ControlNet conditioning and LoRA adaptation) requires approximately 10 seconds per image at 512×512 resolution.

Training of the LoRA models is relatively lightweight, with each model requiring a single training run of approximately 30 minutes. Similarly, the downstream segmentation model is efficient to train, requiring around 10 minutes per run.

It should be noted that these timings are hardware-dependent, particularly on GPU type. Therefore, the reported values should be interpreted as indicative rather than absolute. Nonetheless, they demonstrate that the proposed approach is computationally practical, even in relatively constrained settings.

### 2.8 Downstream segmentation task

A downstream semantic segmentation task is used to evaluate the usefulness of the synthetic data. A U-Net model is trained and assessed across five data configurations: real-only, Stable Diffusion-only, Blender-only, and two hybrid settings combining real data with synthetic samples from Stable Diffusion and Blender.

For the real-only baseline, standard, basic data augmentations, outlined in **Table 3**, are applied to mitigate overfitting on the limited dataset, with transformations chosen to reflect the variability observed in the restricted real data distribution. For experiments involving synthetic data, 100 images are generated and combined with 10 real images per disease, yielding a total training set of 330 images. An additional 15 (5 per disease) are used for validation and a fixed held-out real test set of 30 images (10 per disease) are used for all reported results.

**Table 3:** Basic data augmentations applied to real-only images in the downstream segmentation baseline.

Augmentation	Parameter
Random rotation	$\pm 15^{\circ }$
Random translation	±5% (X), ±10% (Y)
Random scaling	0.95– 1.05×
Horizontal flip	p=0.5
Brightness adjustment	0.7– 1.3×
Gaussian blur	p=0.2, σ=0.1– 0.5
Gaussian noise	σ=0.01

Each model is trained for 20 epochs and then evaluated using the Dice score which is particularly sensitive to small structures, making it well suited for evaluating performance on fine disease lesions. This process is repeated five times for each training fold.

### 2.9 Image distribution similarity

To help evaluate the effectiveness of the style-transfer stage from a numerical perspective, Kernel Inception Distance (KID) (Bińkowski et al., 2018) is used to measure how closely the distributions of synthetic images align with that of the real data. KID computes the Maximum Mean Discrepancy (MMD) between feature embeddings using a pretrained AlexNet (Krizhevsky et al., 2012), selected due to its lower computational cost and stable feature representations when operating on smaller datasets.

For each disease class, 100 generated synthetic images from Blender and the corresponding Stable Diffusion outputs are compared against 100 real images per disease sampled from the PlantVillage dataset. Computing KID on both Blender and Stable Diffusion outputs enables insight into whether the style-transfer process reduces the distributional gap between synthetic and real images.

### 2.10 CLIP-based semantic evaluation

To evaluate the semantic effectiveness of the style-transfer stage, CLIP-based metrics are used to assess both text-image and image-image alignment. CLIP image-text similarity (Hessel et al., 2021) is computed using a ViT-B/32 model, selected for its balance between representational quality and computational efficiency on smaller datasets.

Cosine similarity is calculated between image embeddings and disease-specific text prompts for 100 generated synthetic images from Blender, the corresponding Stable Diffusion output and real images. Scores are averaged across the same four prompts per disease:

1.*”A photo of a leaf infected with [disease].”*

2.*”A close-up shot of [disease] symptoms on a leaf.”*

3.*”A diseased plant leaf showing signs on [disease].”*

4.*”A leaf with visible spots and lesions from [disease].”*

Image-image similarity is also evaluated between the real, Blender and Stable Diffusion images. Cosine distances are computed for real-real, real-Blender and real-Stable Diffusion pairs. A Kernel Density Estimation (KDE) plot is applied to the resulting distributions to visually assess whether style-transfer reduces the semantic gap between synthetic and real images.

### 2.11 Ablation

Ablation experiments are conducted to assess the contribution of individual components within the synthetic generation pipeline to downstream segmentation performance. For each ablation, 100 new synthetic images per disease are generated with a single component removed, whilst all other settings are held constant. The same validation and held-out test sets used in the downstream segmentation experiments are reused to enable direct comparison. Ablations are performed individually for the Canny ControlNet, SoftEdge ControlNet, use of an initial image (Blender render) in the Stable Diffusion pipeline and lesion markers during Blender augmentation.

## 3 Results

### 3.1 Qualitative results

**Figure 5:**
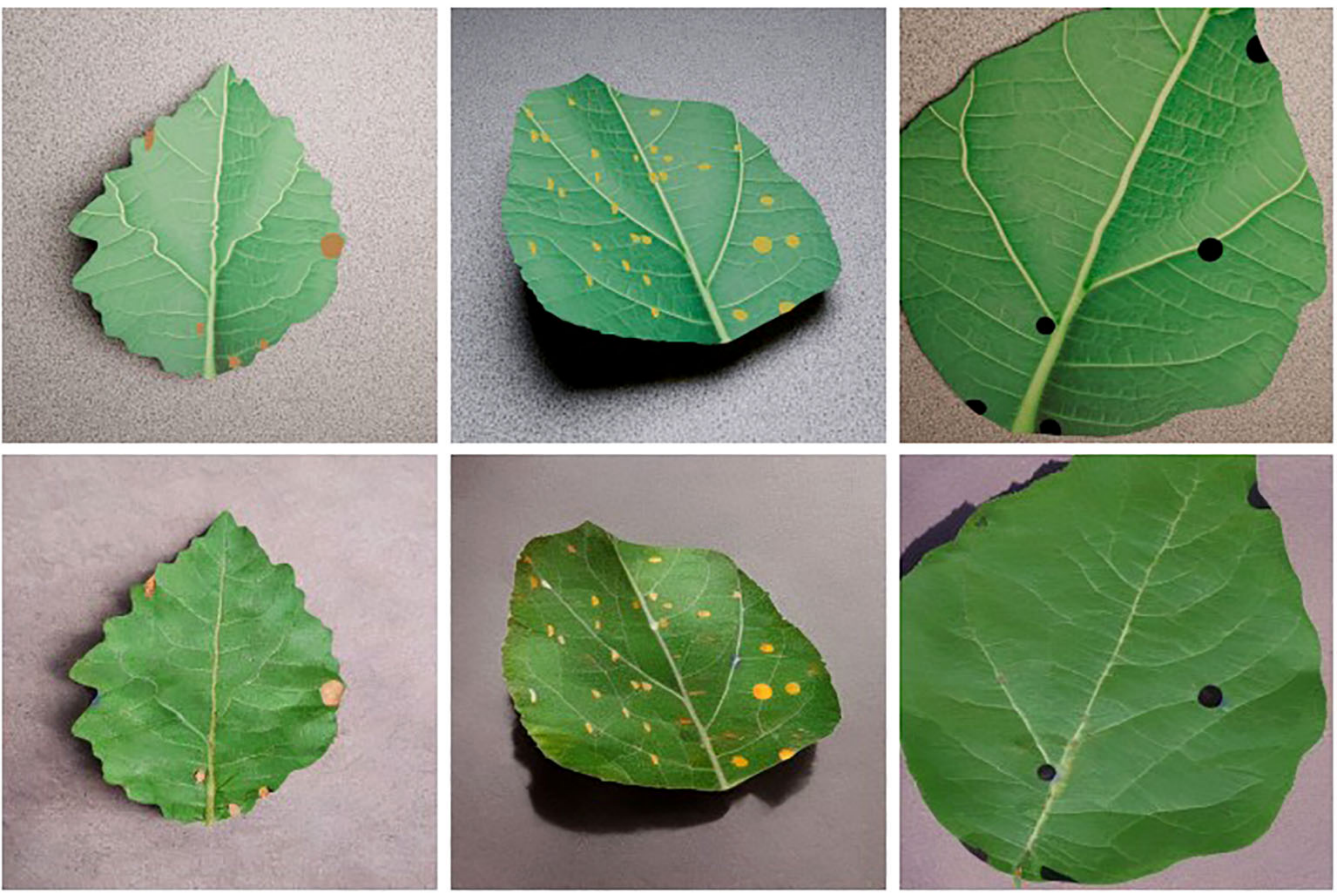
Comparison of Blender renders (top) and diffusion-based style-transfer outputs (bottom), demonstrating preserved structure and enhanced lesion realism.

**Figure 5** illustrates a sample of each disease in the output of the synthetic generation pipeline. Lesion locations and venation patterns are preserved throughout the style-transfer process. Compared to the Blender images, lesions in the Stable Diffusion outputs appear more visually integrated with the leaf surface, exhibiting realistic texture and colour variation. Despite originating from an identical base texture in Blender, the Stable Diffusion samples exhibit variation in colour intensity and surface texture across outputs.

### 3.2 Segmentation results

**Table 4** reports per-class Dice and IoU scores for models trained on real data, synthetic data and a combination of both. Training with a combination of real and synthetic data consistently improves segmentation performance across all classes under both metrics. The largest gains are observed for the disease classes, with black rot improving from 0.27 to 0.76 Dice (0.16 to 0.61 IoU), scab from 0.19 to 0.54 Dice (0.11 to 0.37 IoU) and cedar rust from 0.23 to 0.63 Dice (0.13 to 0.46 IoU). Results are averaged over five runs; however, due to the limited dataset size, no formal statistical significance testing is performed.

**Table 4:** Per-class dice and IoU segmentation results.

Class	Real		Blender		Real + blender		Stable diffusion		Real + stable diffusion	
	Dice	IoU	Dice	IoU	Dice	IoU	Dice	IoU	Dice	IoU
Background	0.95	0.90	0.72	0.56	0.95	0.90	0.83	0.71	**0.95**	**0.90**
Healthy	0.82	0.69	0.52	0.35	0.69	0.53	0.58	0.41	**0.92**	**0.85**
Black rot	0.27	0.16	0.15	0.08	0.31	0.18	0.39	0.24	**0.76**	**0.61**
Scab	0.19	0.11	0.10	0.05	0.10	0.05	0.23	0.13	**0.54**	**0.37**
Cedar rust	0.23	0.13	0.15	0.08	0.21	0.12	0.21	0.12	**0.63**	**0.46**

Bold values indicate the best results with the direction of optimality defined by the table header.

Training on synthetic data alone achieves reasonable performance for the background and healthy classes, but remains substantially weaker on disease classes. This is expected, as background and healthy regions represent simpler segmentation tasks. However, synthetic data still provides some benefit for disease classes, particularly for black rot and scab, where improvements over real-only training are observed.

### 3.3 Image distribution similarity results

**Table 5** reports KID scores for images generated using Blender and Stable Diffusion. This is a way of measuring how similar to the real images the synthetic images appear, in style. Across all disease classes, Stable Diffusion achieves lower (i.e. better) KID values than Blender alone, with the largest reductions observed for black rot and cedar rust, where KID decreases by more than 60%.

**Table 5:** Kernel Inception Distance (KID) between synthetic and real images.

KID score (↓)		
Disease	Blender	Stable diffusion
Black rot	0.23 ± 0.01	**0.12 ± 0.01**
Cedar rust	0.17 ± 0.01	**0.11 ± 0.01**
Scab	0.24 ± 0.01	**0.14 ± 0.01**

Bold values indicate the best results with the direction of optimality defined by the table header.

### 3.4 CLIP-based semantic results

**Table 6** reports the mean CLIP text–image similarity scores for Blender, Stable Diffusion and real images. This captures whether the synthetic images have the same *meaning*, in the form of a textural description, as the real images. Stable Diffusion achieves higher similarity scores (better performance) than Blender alone, improving from 0.30 to 0.33 for scab, from 0.29 to 0.33 for black rot and from 0.29 to 0.32 for cedar rust. To note, in all cases, Stable Diffusion scores approach those of real images.

**Table 6:** Mean CLIP text–image similarity scores for Blender, Stable Diffusion and real images.

Mean text-image CLIP score (↑)			
Disease	Blender	Stable diffusion	Real
Black rot	0.2923	**0.3271**	0.3300
Cedar rust	0.2899	**0.3213**	0.3216
Scab	0.3042	**0.3349**	0.3398

Bold values indicate the best results with the direction of optimality defined by the table header.

**Figure 6:**
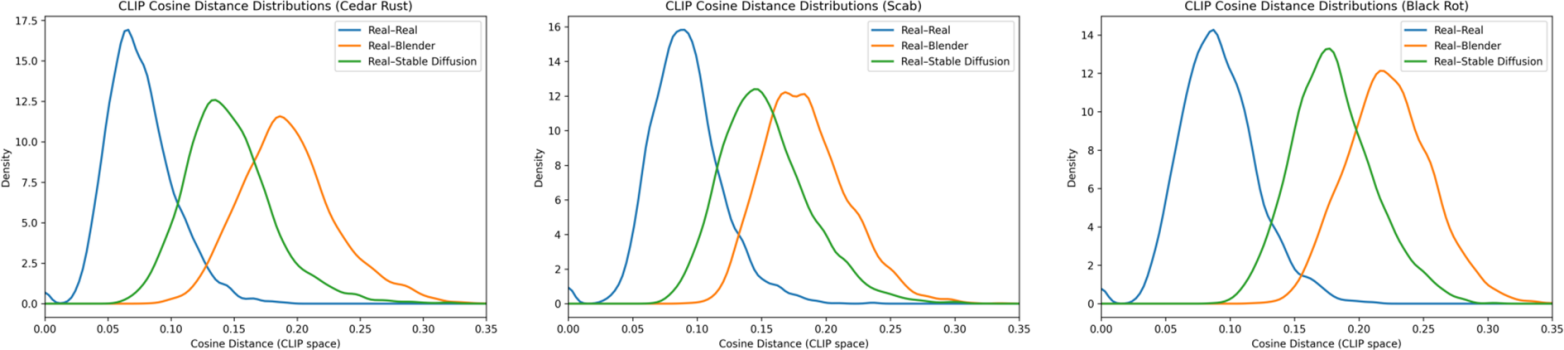
Kernel density estimates of CLIP cosine distance distributions for cedar rust, scab and black Rot comparing real-real, real-Blender and real-Stable Diffusion pairs. Leftward-trending peaks denote stronger semantic similarity to real images, with rightward shifts indicating greater distributional deviation.

KDE plots in **Figure 6** show the distribution of CLIP cosine distances between synthetic and real images within each disease class. Lower cosine distance indicates greater visual similarity. Across all classes, Stable Diffusion images are shifted towards lower distances compared to Blender renders, indicating closer visual alignment with real images. Cedar rust and scab show a similar leftward shift, although with broader distributions and lower peak density than black rot. Among the three diseases, black rot exhibits the closest alignment to the real–real distribution.

### 3.5 Ablation testing results

**Table 7** reports the change in Dice score relative to the full pipeline when individual components are removed. Removing any component generally results in a reduction in Dice score across classes, with the largest degradations observed for the disease classes. The most consistent performance drops occur when removing the SoftEdge or Canny ControlNet, whilst removing the marker constraint produces only minor changes. Removing the initial image results in substantial Dice score reductions across all disease classes.

**Table 7:** Change in Dice score relative to the full pipeline under individual component ablations.

		Δ Dice score (↑)		
Class	– SoftEdge	– Canny	– Lesion markers	– Initial image
Background	-0.0690	-0.0948	+0.0113	+0.0184
Healthy	-0.1109	-0.1491	+0.0160	+0.0100
Black rot	-0.1098	-0.0915	-0.0302	-0.2725
Scab	-0.0868	-0.1202	-0.0340	-0.2452
Cedar rust	-0.0459	-0.0708	-0.0127	-0.2279

**Figure 7:**
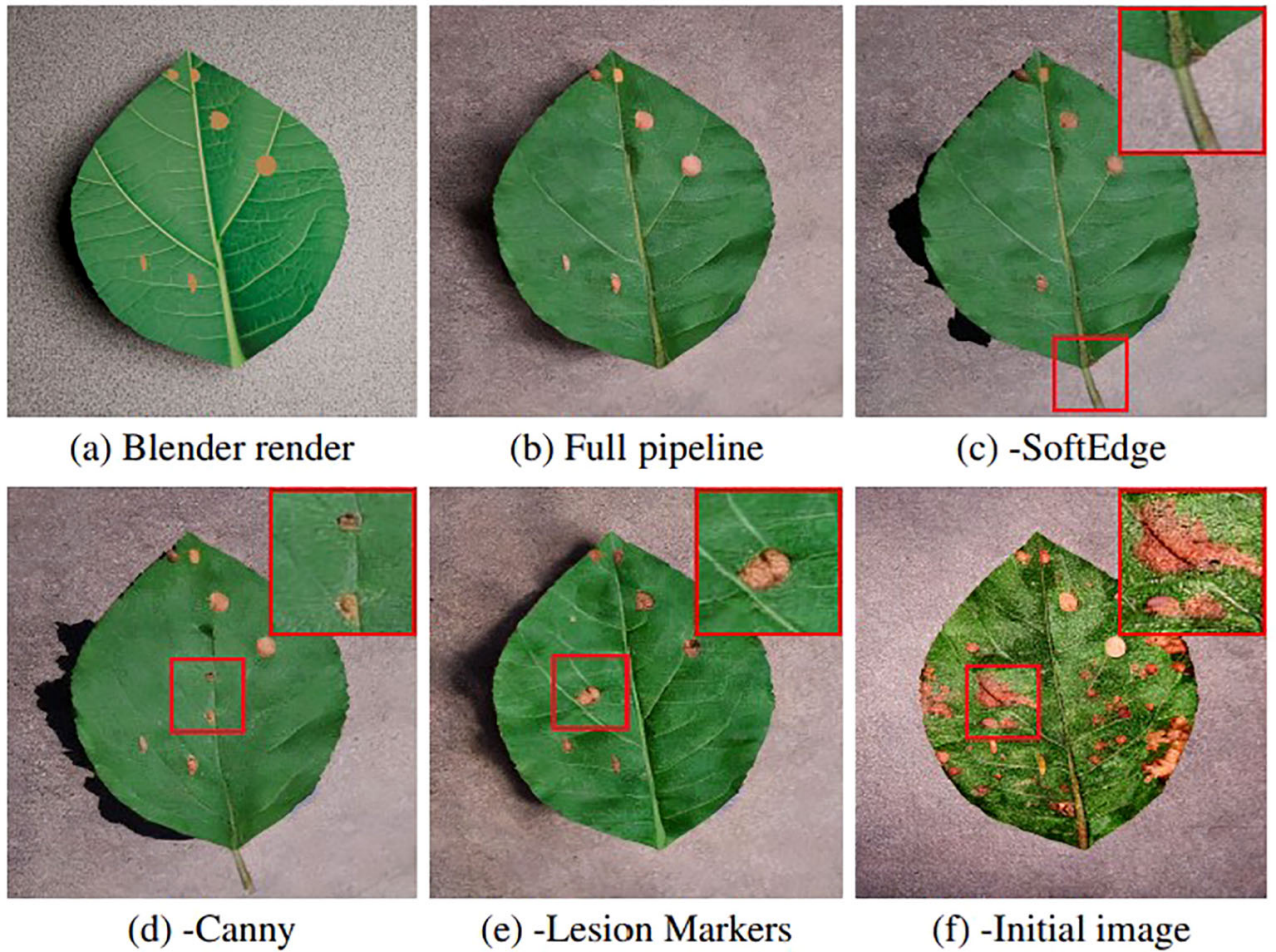
Qualitative ablation comparison with representative errors not limited to specific ablations. **(a)** The original Blender render provides the structural reference. **(b)** The full pipeline maintains correct lesion placement, venation structure and overall geometry. **(c)** Removing SoftEdge introduces an unintended petiole and misplaces lesions relative to the target regions. **(d)** Removing Canny reduces visible venation structure and results in incorrect lesion placement. **(e)** Removing lesion markers leads to additional, unintended lesions beyond the specified locations. **(f)** Removing the initial image produces largely random lesion placement and substantial changes in surface texture. Zoom insets (red border) highlight sample deviations from the Blender render.

**Figure 7** provides a qualitative comparison of generated images under each ablation setting. The full pipeline produces visually coherent leaves with lesions that remain spatially aligned with the Blender render. When SoftEdge or Canny ControlNet is removed, artefacts such as an inconsistent petiole or increased inaccuracy in lesion placement is visible. Removing the lesion markers from the Blender render results in increased variability in lesion placement relative to the original Blender geometry. Removing the initial image produces the largest deviation, with lesions much more dispersed and higher variation in severity.

## 4 Discussion

### 4.1 Downstream segmentation performance

The observed improvements when combining real and Stable Diffusion data suggest that the synthetic images primarily act to regularise the segmentation model rather than replacing real samples. The relatively poor performance of synthetic-only training highlights important limitations of the proposed pipeline. Although diffusion-refined images improve visual realism, they still exhibit a residual domain gap from real data, particularly in fine-scale texture, morphology (e.g. curling or holes) and illumination differences. As a result, models trained solely on synthetic data fail to generalise to real images, especially for small and irregular lesion regions. This is most evident in disease classes, where accurate boundary delineation is critical. Additionally, synthetic data may lack the full variability of real-world disease presentation, leading to overfitting to artefacts specific to the generation process. These findings suggest that synthetic data is most effective as a complementary augmentation rather than a replacement for real data, reinforcing the importance of hybrid training strategies.

### 4.2 Effectiveness of diffusion-based style-transfer

The preservation of lesion and leaf geometry observed in **Figure 5** indicates that strong structural conditioning via Canny and SoftEdge ControlNet effectively constrains the diffusion process, preventing spatial drift during style-transfer. This is important for disease segmentation, where even small misalignments between visual appearance and pixel-level annotations can degrade model performance.

In contrast to Blender renders, which are limited by procedural textures, diffusion-based generation introduces realistic variation in lesion colour, texture and boundary irregularity. This increased appearance diversity is quantitatively reflected by the substantial reduction in KID scores across all disease classes, indicating closer alignment with real image distributions. The corresponding increase in CLIP text-image similarity suggests that these improvements are not just distributional based but reflect more semantically aligned disease representations. CLIP image-image similarity further supports this, showing that Stable Diffusion outputs lie closer to a real image in embedding space than Blender renders, indicating that style-transfer is successful in reducing the semantic gap between synthetic and real imagery. While KID and CLIP provide useful proxies for visual and semantic similarity, they do not directly measure biological correctness or downstream utility. Therefore, these metrics are used as supporting evidence, with segmentation performance forming the primary basis for evaluating effectiveness.

These results directly address the role of diffusion-based refinement within the pipeline. Comparing Real + Blender and Real + Stable Diffusion demonstrates a consistent and substantial improvement across all disease classes when diffusion-refined images are used, confirming that gains are not attributable to synthetic data alone but specifically to the diffusion stage. While Blender-only augmentation provides limited benefit and in some cases degrades performance, the inclusion of diffusion-refined samples leads to large increases in Dice and IoU, particularly for minority disease classes. This establishes a clear link between improved visual/semantic realism and downstream segmentation utility, showing that the diffusion process produces not only more realistic images (as indicated by KID and CLIP), but also more informative supervisory signals. Furthermore, the comparison between Stable Diffusion and Real + Stable Diffusion highlights that synthetic data alone is insufficient, but becomes highly effective when combined with real samples, reinforcing the necessity of the full hybrid pipeline.

### 4.3 Pipeline ablation analysis

Ablation results, outlined in **Table 7**, show that structural constraints are important to segmentation performance. Removing either Canny or SoftEdge ControlNets leads to consistent reductions in disease Dice Scores of up to 0.12. This effect is also qualitatively noticeable in **Figure 7**, where some lesions are either not generated or indeed are neglected. Furthermore, the introduction of new artefacts such as a stem or reduced venation patterns are also noticeable.

Removing disease markers results in a smaller but consistent reduction in disease Dice scores, indicating that lesion appearance and placement can partially emerge from the learned disease prior. However, the qualitative results show increased spatial variability in lesion location without markers, suggesting that they provide a useful additional guide.

The largest performance degradation occurs when the initial image is removed, with Dice reductions exceeding 0.22 across all disease classes. As illustrated in **Figure 7**, the absence of an initial image leads to drastic spatial drift in lesion placement and increased variation in disease severity. This suggests that, without a global structure reference, the diffusion model prioritises visual plausibility over correct structure.

Across all ablation comparisons, the full pipeline demonstrates the strongest visual consistency with the Blender render, preserving both lesion placement and underlying venation structure. Notably, the shadowing in the full pipeline appears more natural than in several constrained variants, suggesting that reducing conditioning constraints can limit the diffusion model’s ability to generate realistic illumination under identical generation parameters.

These results show that diffusion-based augmentation for segmentation must prioritise spatial conditioning over unconstrained realism. For example, whilst visually plausible outputs can be produced without an initial image, the resulting loss of correspondence directly degrades segmentation accuracy.

### 4.4 Comparison to prior synthetic pipelines

Prior work has applied diffusion-based style-transfer to synthetic plant imagery in settings where preserving overall leaf morphology is sufficient for downstream segmentation (Hartley et al., 2024). In contrast, disease segmentation requires precise localisation of small, irregular lesions, making performance highly sensitive to even minor spatial drift. Our ablation results indicate that this added complexity necessitates stronger geometric constraints during generation, highlighting a task-dependent trade-off between visual realism and spatial control that is less critical in whole-leaf segmentation.

Blender-only synthetic datasets used for mushroom (Anagnostopoulou et al., 2023) or strawberry (Aghamohammadesmaeilketabforoosh et al., 2025) segmentation achieve strong results for background and foreground separation under controlled conditions. In these tasks, the target structures are geometrically simple and can be modelled using procedural shape and texture variation. In contrast, plant diseases exhibit irregular boundaries, colour variation and surface integration that are difficult to reproduce using rendering alone. The KID and CLIP results show that diffusion-based style-transfer is required to capture this appearance complexity, reducing the gap to real diseased imagery.

### 4.5 Limitations

While the results were promising, there are several limitations of the proposed approach. A key trade-off exists between structural control and visual realism. Strong ControlNet guidance is required to preserve lesion placement and pixel-level alignment, however, this can restrict appearance variation and occasionally lead to less visually natural outputs. This trade-off was a deliberate design choice, prioritising mask alignment for segmentation over unconstrained realism, but may limit applicability in tasks where exact correspondence is not required.

Lesion placement in the current pipeline does not model disease-specific spatial patterns. In real leaves, certain diseases exhibit preferential localisation, such as clustering along veins or near the stem, which is not explicitly captured by the uniformly random placement strategy used in this work. Incorporating biologically informed spatial priors, for example distance-to-vein maps or learned placement distributions, could further improve realism and downstream performance.

Disease severity is not explicitly controlled. While the pipeline introduces variation in lesion appearance, controlling the extent and progression of disease remains challenging, limiting the ability to generate samples across a continuous severity spectrum. Future work could address this through conditional generation mechanisms, such as severity tokens or explicit constraints on lesion count and coverage.

All stages of the pipeline, including LoRA fine-tuning and 3D modelling, were conducted using the PlantVillage dataset, which contains images captured under controlled conditions with minimal background complexity. As a result, segmentation performance is unlikely to generalise to in-field imagery with variable lighting, occlusion and background clutter. Extending the pipeline to incorporate background variation or mixed-domain training would be necessary to address this domain gap. Furthermore, the small dataset size limits the statistical strength of conclusions, and results should be interpreted as indicative rather than definitive.

### 4.6 Conclusion

This work presents a synthetic data generation pipeline combining 3D leaf modelling with diffusion-based style-transfer for plant disease segmentation in low-resource settings. Practicality is demonstrated by integrating synthetic samples with limited real data, improving downstream segmentation performance across disease classes. Results indicate that diffusion-based refinement reduces the domain gap relative to 3D rendering alone, whilst ablation experiments show that structural constraints via ControlNet guidance and an initial image contribute to improved pixel alignment and segmentation accuracy. Overall, the pipeline provides an effective approach for augmenting limited datasets within controlled settings.

## Data Availability

The datasets presented in this study can be found in online repositories. The names of the repository/repositories and accession number(s) can be found in the article/supplementary material.

## References

[B1] Aghamohammadesmaeilketabforoosh K. Parfitt J. Nikan S. Pearce J. M. (2025). From blender to farm: Transforming controlled environment agriculture with synthetic data and swinunet for precision crop monitoring. PloS One 20, 1–17. doi: 10.1371/journal.pone.0322189 40273145 PMC12021149

[B2] Anagnostopoulou D. Retsinas G. Efthymiou N. Filntisis P. Maragos P. (2023). “ A realistic synthetic mushroom scenes dataset”, in: 2023 IEEE/CVF Conference on Computer Vision and Pattern Recognition Workshops (CVPRW) (New York, USA: IEEE (Institute of Electrical and Electronics Engineers)), 6282–6289. doi: 10.1109/CVPRW59228.2023.00668

[B3] Barbedo J. (2019). Plant disease identification from individual lesions and spots using deep learning. Biosyst. Eng. 180, 96–107. doi: 10.1016/j.biosystemseng.2019.02.002 38826717

[B4] Ben Salem H. (2024). Tackling domain shift in AI: A deep dive into domain adaptation. Medium.

[B5] Bińkowski M. Sutherland D. Arbel M. Gretton A. (2018). Demystifying mmd gans. doi: 10.48550/arXiv.1801.01401

[B6] Blender Foundation (2024). Blender – 3D modelling and rendering software (Amsterdam, Netherlands: Blender Foundation). Available online at: http://www.blender.org (Accessed February 20, 2026).

[B7] Chen L.-C. Zhu Y. Papandreou G. Schroff F. Adam H. (2018). “ Encoder-decoder with atrous separable convolution for semantic image segmentation”, in: Computer Vision – ECCV 2018 (Cham, Switzerland: Springer), 833–851.

[B8] Dosovitskiy A. Beyer L. Kolesnikov A. Weissenborn D. Zhai X. Unterthiner T. . (2021). “ An image is worth 16x16 words: Transformers for image recognition at scale”, in: International Conference on Learning Representations ( OpenReview / ICLR).

[B9] Fahlgren N. Gehan M. A. Baxter I. (2015). Lights, camera, action: high-throughput plant phenotyping is ready for a close-up. Curr. Opin. Plant Biol. 24, 93–99. doi: 10.1016/j.pbi.2015.02.006 25733069

[B10] Food and Agriculture Organization of the United Nations (2025a). Plant production and protection. Available online at: https://www.fao.org/plant-production-protection (Accesseed December 15, 2025).

[B11] Food and Agriculture Organization of the United Nations (2025b). The state of food and agriculture 2025: Making agri-food systems more resilient to shocks and stresses. Available online at: https://www.fao.org/publications/sofa (Accessed December 15, 2025).

[B12] Furbank R. T. Tester M. (2011). Phenomics – technologies to relieve the phenotyping bottleneck. Trends Plant Sci. 16, 635–644. doi: 10.1016/j.tplants.2011.09.005 22074787

[B13] Goodfellow I. J. Pouget-Abadie J. Mirza M. Xu B. Warde-Farley D. Ozair S. . (2014). “ Generative adversarial nets”, in: Advances in Neural Information Processing Systems (Red Hook, NY, USA: Curran Associates, Inc), 27.

[B14] Hartley Z. K. J. Lind R. J. Pound M. P. French A. P. (2024). “ Domain targeted synthetic plant style transfer using stable diffusion, lora and controlnet”, in: IEEE/CVF Conference on Computer Vision and Pattern Recognition, CVPR 2024 (New York, USA: IEEE), 5375–5383. doi: 10.1109/CVPRW63382.2024.00546

[B15] Hessel J. Holtzman A. Forbes M. Bras R. L. Choi Y. (2021). Clipscore: A reference-free evaluation metric for image captioning. CoRR abs/2104.08718. doi: 10.18653/v1/2021.emnlp-main.595 42035093

[B16] Hu E. J. Shen Y. Wallis P. Allen-Zhu Z. Li Y. Wang S. . (2021). Lora: Low-rank adaptation of large language models. ArXiv abs/2106.09685.

[B17] Huber L. Gillespie T. (2003). Modeling leaf wetness in relation to plant disease epidemiology. Annu. Rev. Phytopathol. 30, 553–577. doi: 10.1146/annurev.py.30.090192.003005 41139587

[B18] Hughes D. P. Salathe M. (2016). An open access repository of images on plant health to enable the development of mobile disease diagnostics. ArXiv 1511.08060. doi: 10.3389/fpls.2016.01419

[B19] Krizhevsky A. Sutskever I. Hinton G. E. (2012). “ Imagenet classification with deep convolutional neural networks”, in: Advances in Neural Information Processing Systems (Red Hook, NY, USA: Curran Associates, Inc.), 25. doi: 10.1145/3065386

[B20] Kumar S. Choudhary M. Reddy J. K. Vishwakarma V. K. Kashyap V. K. Sahoo S. . (2024). A review on the impact of climate change on plant pathogen interactions. J. Adv. Microbiol. 24, 11–27. doi: 10.9734/jamb/2024/v24i8843 39075931

[B21] Mahlein A.-K. (2016). Plant disease detection by imaging sensors – parallels and specific demands for precision agriculture and plant phenotyping. Plant Dis. 100, 241–251. doi: 10.1094/PDIS-03-15-0340-FE 30694129

[B22] Mahlein A.-K. Kuska M. T. Behmann J. Polder G. Walter A. (2018). Hyperspectral sensors and imaging technologies in phytopathology: State of the art. Annu. Rev. Phytopathol. 56, 535–558. doi: 10.1146/annurev-phyto-080417-050100 30149790

[B23] Migicovsky Z. Li M. Chitwood D. H. Myles S. (2018). Morphometrics reveals complex and heritable apple leaf shapes. Front. Plant Sci. 8. doi: 10.3389/fpls.2017.02185 29354142 PMC5758599

[B24] Mohanty S. P. Hughes D. P. Salathé M. (2016). Using deep learning for image-based plant disease detection. Front. Plant Sci. 7. doi: 10.3389/fpls.2016.01419 27713752 PMC5032846

[B25] Polly R. devi E. (2024). Semantic segmentation for plant leaf disease classification and damage detection: A deep learning approach. Smart Agric. Technol. 9, 100526. doi: 10.1016/j.atech.2024.100526 38826717

[B26] Rombach R. Blattmann A. Lorenz D. Esser P. Ommer B. (2022). “ High-resolution image synthesis with latent diffusion models”, in: Proceedings of the IEEE/CVF Conference on Computer Vision and Pattern Recognition (CVPR) (New York, USA: IEEE), 10684–10695.

[B27] Ronneberger O. Fischer P. Brox T. (2015). “ U-net: Convolutional networks for biomedical image segmentation”, in: Medical Image Computing and Computer-Assisted Intervention – MICCAI (Cham, Switzerland: Springer), 234–241. doi: 10.1007/978-3-319-24574-4_28

[B28] von Platen P. Patil S. Lozhkov A. . (2022). Diffusers: State-of-the-art diffusion models. Available online at: https://github.com/huggingface/diffusers.

[B29] Wang C. Xia Y. Xia L. Wang Q. Gu L. (2025). Dual discriminator GAN-based synthetic crop disease image generation for precise crop disease identification. Plant Methods 21, 46. doi: 10.1186/s13007-025-01361-0 40159478 PMC11955132

[B30] Xie S. Tu Z. (2015). “ Holistically-nested edge detection”, in: 2015 IEEE International Conference on Computer Vision (ICCV) (New York, USA: IEEE), 1395–1403. doi: 10.1109/ICCV.2015.164

[B31] Zhang L. Rao A. Agrawala M. (2023). “ Adding conditional control to text-to-image diffusion models”, in: 2023 IEEE/CVF International Conference on Computer Vision (ICCV) (New York, USA: IEEE), 3813–3824. doi: 10.1109/ICCV51070.2023.00355

[B32] Zhou Z. Rahman Siddiquee M. M. Tajbakhsh N. Liang J. (2018). “ Unet++: A nested u-net architecture for medical image segmentation”, in: Deep Learning in Medical Image Analysis and Multimodal Learning for Clinical Decision Support (Cham, Switzerland: Springer), 3–11. doi: 10.1007/978-3-030-00889-5_1 PMC7329239 32613207

[B33] Zou X. Zhang S. Li K. Wang S. Xing J. Jin L. . (2025). Adapting vision foundation models for robust cloud segmentation in remote sensing images. IEEE Trans. Geosci. Remote Sens. 63, 1–14. doi: 10.1109/TGRS.2025.3597410 25079929

