# Correction: Synthetic plant disease image generation to improve segmentation tasks in low-resource settings

**DOI:** 10.3389/fpls.2026.1880531

**Published:** 2026-06-25

**Authors:** Elliott Cooper, Zane Hartley, Andrew French

**Affiliations:** School of Computer Science, University of Nottingham, Nottingham, United Kingdom

**Keywords:** ControlNet, leaf segmentation, low-rank adaptation, low-resource learning, plant disease detection, semantic segmentation, stable diffusion, synthetic data

There was a mistake in **Table 4** as published. Only the two “Real+Stable Diffusion” columns should be bolded, correctly identifying the best performance. The corrected **Table 4** appears below.

**Table 4:** Per-class dice and IoU segmentation results.

Class	Real		Blender		Real + blender		Stable diffusion		Real + stable diffusion	
	Dice	IoU	Dice	IoU	Dice	IoU	Dice	IoU	Dice	IoU
Background	0.95	0.90	0.72	0.56	0.95	0.90	0.83	0.71	**0.95**	**0.90**
Healthy	0.82	0.69	0.52	0.35	0.69	0.53	0.58	0.41	**0.92**	**0.85**
Black rot	0.27	0.16	0.15	0.08	0.31	0.18	0.39	0.24	**0.76**	**0.61**
Scab	0.19	0.11	0.10	0.05	0.10	0.05	0.23	0.13	**0.54**	**0.37**
Cedar rust	0.23	0.13	0.15	0.08	0.21	0.12	0.21	0.12	**0.63**	**0.46**

Bold values indicate the best results with the direction of optimality defined by the table header.

The original version of this article has been updated.

